# α_V_β_3_ Integrin regulates astrocyte reactivity

**DOI:** 10.1186/s12974-017-0968-5

**Published:** 2017-09-29

**Authors:** Raúl Lagos-Cabré, Alvaro Alvarez, Milene Kong, Francesca Burgos-Bravo, Areli Cárdenas, Edgardo Rojas-Mancilla, Ramón Pérez-Nuñez, Rodrigo Herrera-Molina, Fabiola Rojas, Pascal Schneider, Mario Herrera-Marschitz, Andrew F. G. Quest, Brigitte van Zundert, Lisette Leyton

**Affiliations:** 1 Cellular Communication Laboratory, Programme of Cellular & Molecular Biology, Instituto de Ciencias Biomédicas, Facultad de Medicina, Universidad de Chile, 838-0453 Santiago, Chile; 2 Center for Molecular Studies of the Cell (CEMC), Advanced Center for Chronic Diseases (ACCDiS), Facultad de Medicina, Universidad de Chile, 838-0453 Santiago, Chile; 3 Facultad de Ciencia, Universidad San Sebastian, Santiago, Chile; 4 Department of Biomedicine, Faculty of Health Sciences, University of Antofagasta, Antofagasta, Chile; 5 Departamento de Ciencias Químicas y Biológicas, Universidad Bernardo O’Higgins, 837-0854 Santiago, Chile; 6 Leibniz Institute for Neurobiology, Magdeburg, Germany; 7 Center for Biomedical Research, Faculty of Biological Sciences and Faculty of Medicine, Universidad Andres Bello, Santiago, Chile; 8 Department of Biochemistry, University of Lausanne, 1066 Epalinges, Switzerland; 9 Programme of Molecular & Clinical Pharmacology, Instituto de Ciencias Biomédicas, Facultad de Medicina, Universidad de Chile, 838-0453 Santiago, Chile

**Keywords:** Integrins, Inflammation, Reactive astrocytes, Amyotrophic lateral sclerosis, Cell migration

## Abstract

**Background:**

Neuroinflammation involves cytokine release, astrocyte reactivity and migration. Neuronal Thy-1 promotes DITNC1 astrocyte migration by engaging α_V_β_3_ Integrin and Syndecan-4. Primary astrocytes express low levels of these receptors and are unresponsive to Thy-1; thus, inflammation and astrocyte reactivity might be necessary for Thy-1-induced responses.

**Methods:**

Wild-type rat astrocytes (TNF-activated) or from human SOD1^G93A^ transgenic mice (a neurodegenerative disease model) were used to evaluate cell migration, Thy-1 receptor levels, signaling molecules, and reactivity markers.

**Results:**

Thy-1 induced astrocyte migration only after TNF priming. Increased expression of α_V_β_3_ Integrin, Syndecan-4, P2X7R, Pannexin-1, Connexin-43, GFAP, and iNOS were observed in TNF-treated astrocytes. Silencing of β_3_ Integrin prior to TNF treatment prevented Thy-1-induced migration, while β_3_ Integrin over-expression was sufficient to induce astrocyte reactivity and allow Thy-1-induced migration. Finally, hSOD1^G93A^ astrocytes behave as TNF-treated astrocytes since they were reactive and responsive to Thy-1.

**Conclusions:**

Therefore, inflammation induces expression of α_V_β_3_ Integrin and other proteins, astrocyte reactivity, and Thy-1 responsiveness. Importantly, ectopic control of β_3_ Integrin levels modulates these responses regardless of inflammation.

**Electronic supplementary material:**

The online version of this article (10.1186/s12974-017-0968-5) contains supplementary material, which is available to authorized users.

## Background

Astrocytes are the main type of glial cell in the central nervous system (CNS). Their functions range from nursing neurons to forming the blood-brain barrier. Under pro-inflammatory conditions induced by injury or neurodegenerative disorders, such as Alzheimer’s disease and amyotrophic lateral sclerosis (ALS), astrocytes become reactive and take on a more stellate shape, while increasing in number and size. At the same time, the expression of several proteins is enhanced, including that of glial fibrillary acidic protein (GFAP) and inducible nitric oxide synthase (iNOS) [1–5]. This reactive process is referred to as astrogliosis [6–9]. Astrogliosis has been recognized as an inhibitory cue for axon regeneration. Nevertheless, it may have initial benefits for the injured area, since astrocytes restrict any damage to a small area, repair the blood brain barrier, and eliminate toxic factors from the surrounding area [8–10]. During astrogliosis, several molecules are released into the extracellular space, including ATP, adenosine, and a number of cytokines [8, 9]. One of the most important cytokines released to the injured area is tumor necrosis factor (TNF) [9, 11], which acts as an alert signal that induces reactivity of the neighboring astrocytes [12, 13].

Reactive astrocytes are a heterogeneous population of cells, and recent in vivo studies indicate that some astrocytes proliferate, while for another subset of labeled astrocytes hypertrophy and polarization, but not overt migration towards the site of injury are detected [14–16]. However, several previously reported studies indicate that reactive astrocytes migrate to the damaged zone and form the glial scar, repairing the wounded area [6, 10, 17]. The protein osteopontin is among the various stimuli that induce cell migration; this requires interaction with α_V_β_3_ Integrin [18, 19]. Importantly, reactive astrocytes have been shown to express increased levels of α_V_β_3_ Integrin, the P2X7 receptor (P2X7R), and the hemichannels Connexin-43 and Pannexin-1 [4, 18, 20]. All these molecules are reportedly involved in astrocyte migration [21, 22].

The neuronal surface glycoprotein Thy-1 binds to astrocytes and thereby promotes adhesion and migration, processes that our group has studied in depth. Thy-1-induced adhesion and migration require interaction with the α_V_β_3_ Integrin and Syndecan-4 receptors. These interactions trigger the recruitment of focal adhesion (FA) proteins and activate a signaling module, which in turn leads to the activation of the small GTPase RhoA and its effector ROCK and results in changes in the astrocyte actin cytoskeleton [23–28]. The signaling cascade also includes the opening of the hemichannels Connexin-43 and Pannexin-1 and the release of ATP to the extracellular milieu, where it activates P2X7R, allowing calcium entry [21, 22]. After a more prolonged stimulation, Thy-1 induces the activation of FAK, PI3-kinase, and the GTPase Rac1, thus leading to astrocyte migration [26].

We have previously studied the Thy-1-induced signaling cascade using the DITNC1 astrocyte cell line. Considering that, in primary astrocytes, many of the molecules forming part of the Thy-1-induced signaling pathway are only present in low amounts [29, 30], we hypothesized that primary astrocytes would be refractory to Thy-1 stimulus. Additionally, many signaling proteins, including the Thy-1 receptors, are upregulated under pro-inflammatory conditions [3, 4, 18, 20, 30]. We therefore decided to directly study the effect of a pro-inflammatory environment on the response of primary astrocytes to Thy-1, and on the Thy-1-induced signaling cascade in these cells. This was achieved in two ways. Firstly, primary astrocytes were treated with the pro-inflammatory signaling factor TNF prior to Thy-1 application, comparing the results to those obtained from controls that had not been exposed to TNF. Secondly, astrocytes were used derived from an ALS model, a neurodegenerative disorder marked by neuroinflammation and the presence of reactive astrocytes [31].

ALS is a late-onset disease, which generally appears after 50 years of age. It is characterized by motor neuron dysfunction and degeneration and leads to death from respiratory failure [32–34]. Astrocytes from ALS patient samples are activated [35] and show increased levels of Connexin-43 and GFAP [3, 36]. In murine ALS models, astrocytes are activated within a time frame before the onset of disease [31, 37]. Additionally, these reactive astrocytes are now recognized to assist ALS motor neuronal death by secreting inflammatory factors [33, 36, 38–42]. To study the effect of Thy-1 signaling on astrocytes developing under the ALS neuroinflammatory conditions, the transgenic mouse carrying the human G93A mutated super oxide dismutase 1 (hSOD1^G93A^) was employed, the most widely used ALS model [1, 3, 33, 36, 39, 41–43].

As discussed above, Thy-1-mediated astrocyte adhesion and migration relies on the α_V_β_3_ Integrin receptor. Because β_3_ Integrin is upregulated in the presence of TNF in various cell types [18, 44, 45], it could be hypothesized that the effect of TNF on astrocyte reactivity is related to β_3_ Integrin expression. To further explore the role of this receptor molecule, Thy-1 signaling in primary astrocytes was therefore investigated, in which β_3_ Integrin expression had been either silenced or upregulated, both in the presence and absence of TNF.

Here, we show that primary astrocytes only responded to Thy-1 in a pro-inflammatory environment (+TNF), where proteins participating in the Thy-1 response were upregulated. In addition, neonatal primary astrocytes derived from a mouse model of ALS (hSOD1^G93A^) were found to express higher levels of reactivity markers and β_3_ Integrin, Syndecan-4, Connexin-43, and Pannexin-1 than those derived from wild-type non-transgenic littermates. The hSOD1^G93A^-derived astrocytes responded to Thy-1 without the need of cytokine addition. Importantly, in investigating the role of β_3_ Integrin in Thy-1-induced FA and astrocyte migration, over-expression of β_3_ Integrin was found to be sufficient to render primary astrocytes reactive and consequently responsive to Thy-1 stimulation in the absence of TNF. Conversely, silencing of β_3_ Integrin precluded the effect of Thy-1 on TNF-treated astrocytes. These results favor a working model in which inflammation enhances β_3_ Integrin expression, rendering astrocytes responsive to Thy-1 and thereby promoting their adhesion and migration, as required during the repair of the injured brain. Controlling β_3_ Integrin levels modulated astrocyte reactivity.

## Methods

### Animals

The care and use of rodents is detailed in protocols approved by the bioethical Committees of the Universidad de Chile and Universidad Andres Bello. Wistar neonatal rats (P1-P2) were obtained from the Universidad de Chile animal facility. Transgenic mice hemizygous for human SOD1^G93A^ (hSOD1^G93A^) or non-transgenic mice (used as a control) were obtained from the Jackson Laboratory (Bar Harbor, USA). Transgenes were detected by PCR [39].

### Primary cultures

Astrocytes were obtained from 1- to 2-day-old wild-type rats or non-transgenic and hSOD1^G93A^ mice. Briefly, wild-type rats were sacrificed by decapitation. Brains were extracted and placed in ice-cold PBS under a microscope. Cortices were separated from brain and meninges and kept in fresh ice-cold PBS. To isolate the cells, cortices were treated with trypsin and mechanically disrupted. The resulting cell suspensions were passed through a 70-μm cell strainer, washed with DMEMF-12 (Gibco, Life Technologies, Grand Island, NY) supplemented with 10% FBS, and centrifuged at 1000 rpm. Following this, cells were counted and seeded at a density of 1.5 million cells per T-25 cell flask. The cell medium was changed every 48 h. Once confluent, neurons and other cell types were released to the supernatant by shaking in an orbital shaker at 180 rpm overnight. To induce a pro-inflammatory environment, rat primary astrocytes were stimulated with 10 ng/ml of TNF for 48 h. IL-1β, IL-6, or IFN-γ (R&D systems) were also used (kindly donated by Dr. Rommy von Bernhardi, P. Universidad Católica de Chile). Murine astrocyte cultures were prepared from the ventral spinal cord of P1-P2 hSOD1^G93A^ transgenic mice, or from non-transgenic littermates as previously described [39, 41, 42]. Cultures were maintained in DMEM (Gibco, Life Technologies, Grand Island, NY) containing 10% FBS and 1% penicillin-streptomycin at 37 °C, 5% CO_2_. Cultures reached confluence after 2–3 weeks and contained > 95% GFAP^+^ astrocytes. Residual microglia were removed from both mouse and rat astrocytes by shaking cultures in an orbital shaker overnight (200 rpm).

### Thy-1-Fc and Trail-R2-Fc preparation

Thy-1-Fc (wild-type and mutants) and Trail-R2-Fc fusion proteins were obtained as previously described [21, 26]. Prior to their use, Thy-1-Fc and Trail-R2-Fc were incubated with Protein A in a 10:1 ratio wile rotating gently on a shaker for 1 h at 4 °C. Trail-R2-Fc is a fusion protein of the receptor for the soluble apoptosis-inducing ligand Trail-R2, which is used as a control for possible non-specific effects caused by the Fc portion of the Thy-1 fusion protein [21]. Prior to each experiment involving Thy-1-Fc or Trail-R2-Fc treatment, astrocytes were serum-starved for at least 30 min in DMEM without serum.

### Focal adhesion assay

Primary astrocytes were seeded on 12-mm coverslips and left to adhere for 24 h. Cells were then pre-treated with 10 ng/ml of TNF for 48 h to induce reactivity. Afterwards, astrocytes were rinsed with DMEM and left in serum-free medium for 30 min. This medium was then removed, and cells were incubated for 15 min with 4 μg of either Thy-1-Fc or Trail-R2-Fc coupled to Protein A in serum-free medium. FBS (3% for 5 min) was used as a positive control. Astrocytes were then washed and fixed for 10 min with 4% paraformaldehyde, permeabilized with 0.1% Triton X-100 for 10 min, and blocked in 2% bovine serum albumin (BSA)-PBS for 1 h. Cells were then incubated with anti-vinculin antibody (1:200, Sigma-Aldrich Co., Saint Louis, MO) for 1 h at 37 °C to stain for focal adhesions. Rhodamine-conjugated Phalloidin (Sigma-Aldrich Co., Saint Louis, MO) and DAPI (Sigma-Aldrich Co.) (0.025 μg/ml) were used to stain F-actin and nuclei, respectively. Quantification of focal adhesion area and number was performed as described previously [21, 26].

### Transfection of primary astrocytes

Astrocytes were transfected with pEGFP-β_3_ Integrin (full length β_3_ Integrin subunit kindly donated by Dr. C. Rüegg, University of Friboug, Switzerland [46]) or a pool of three siRNAs against β_3_ Integrin (Ambion) using the Amaxa Nucleofector system following the manufacturer’s instructions for the VPI-1006 transfection kit and the program T-020 (Lonza, Cologne, Germany).

### Wound-healing assay

Primary astrocytes were seeded in 24-well plates (100,000 cells per well). After 48 h of incubation in complete medium with or without TNF (10 ng/ml), two parallel wounds were introduced with a micropipette tip. Detached cells were washed away with serum-free medium, and after 30 min of starvation, cells were stimulated with 4 μg of Thy-1-Fc, Thy-1(RLE)-Fc, or Trail-R2-Fc (negative control) in serum-free medium for 24 h. All proteins were coupled to Protein A as indicated above. Alternatively, primary astrocytes were transfected with pEGFP-β_3_ Integrin or siRNA against β_3_ Integrin, prior to wounding cell monolayers and stimulating as above without TNF pre-treatment. Wound healing was evaluated based on images of the cell-free area at 0 and 24 h, as previously described [21, 26].

### Western blot

Protein extracts were prepared in a lysis buffer (150 mM NaCl, 0.1% SDS, 0.25% sodium deoxycholate, 1% Triton-X100, in 50 mM Tris-HCl pH 7.4) supplemented with protease and phosphatase inhibitor cocktail (Biotool, Houston, TX). Extracts were electrophoretically separated on 10% SDS-PAGE gels and transferred to nitrocellulose (Millipore, Billerica, USA). The nitrocellulose was blocked with 5% *w*/*v* nonfat, dry milk in PBS containing 0.1% Tween-20 and subsequently incubated with the following primary antibodies: anti-β_3_ Integrin (1:2000, Millipore, Billerica, USA), anti-Syndecan-4 (1:2000, Abbexa, Cambridge, UK), anti-iNOS (Abbexa), anti-Connexin-43 (1:500; Santa Cruz Biotechnologies Dallas, TX), anti-Pannexin-1 (1:500; Santa Cruz Biotechnologies), anti-P2X7R (1:1000, Santa Cruz Biotechnologies), anti-GFAP (1:2000, Sigma-Aldrich Co.), and anti-misfolded human SOD1 (C4F6) (MediMabs, Quebec, Canada). The membrane was then washed and incubated with horseradish peroxidase-conjugated goat anti-rabbit IgG (1:5000; Abbexa, Cambridge, UK) or donkey anti-goat IgG (1:5000; Abbexa) for 1 h at room temperature. Bands were visualized with a chemiluminescence kit (Pierce, Thermo Scientific, Rockford, IL), according to the manufacturer’s instructions. We performed Western blot quantification by measuring band intensity in Photoshop 7 (Adobe) and normalizing to the corresponding loading control. To determine fold increase, all values were normalized to the average value obtained for control bands after any treatment.

### Indirect immunofluorescence assay

Astrocytes were seeded on 12-mm coverslips and left to adhere for 24 h. Cells were then washed and fixed as indicated for the focal adhesion assay. Next, cells were stained with anti-Connexin-43 (1:200), anti-Pannexin-1 (1:200), anti-Syndecan-4 (1:200) or anti-P2X7R (1:200) antibodies followed by secondary antibodies (1:400) conjugated to IF488 or IF594 (Abbexa), and DAPI (0.025 μg/ml). Samples were analyzed using a confocal microscope C2+ (Nikon, Japan), with a 60×/1.40 objective and using the NIS-Elements software.

### Extracellular ATP measurements

Twelve thousand primary astrocytes were seeded in 48-well plates. After 24 h, cells were incubated in serum-free media containing 100 μM of the exonuclease inhibitor ARL-67156 (Santa Cruz Biotechnologies) for 30 min at 37 °C. Following this, cells were stimulated with Thy-1-Fc:Protein A for 15 min. Where indicated, cells were incubated with a combination of Heptanol (Sigma-Aldrich Co.) (500 μM) and Probenecid (Sigma-Aldrich Co.) (1 mM). Next, culture medium was recovered and centrifuged for 5 min at 1000×*g*. The supernatants were incubated in the dark with 50 μl CellTiter-Glo® reaction mix (Promega, Madison, WI). Luminescence intensity was determined in a Synergy2 multi-mode reader (Biotek Instruments, Inc., Vermont), and the values were interpolated using a calibration curve obtained from different ATP concentrations (0.01, 0.1, 1, and 10 μM).

### Measurement of intracellular calcium kinetics

Primary astrocytes were seeded on 25-mm coverslips (400,000 cells) and left to adhere for 24 h. The cells were then incubated with 5 μM Fluo-4-AM (Life Technologies, Grand Island, NY) in Ringer solution at 37 °C for 30 min, after which they were washed and left in the same solution. Following this, astrocytes were treated with the hemichannel blockers Heptanol (500 μM) and Probenecid (1 mM) for 30 min. Alternatively, they were treated with the P2X7R blocker BBG (American Bioanalytical, Natick, MA) (5 μM for 1 h). Images were acquired at 2-s intervals with an XM10 camera (Olympus Corp.). Thy-1-Fc or Trail-R2-Fc, both coupled to Protein A, were added 120 s after initiating acquisition. Fluorescence intensity was quantified in 100 cells per condition using ImageJ. The results were expressed as (F-F0)/F0, where F is the change in fluorescence and F0 is the basal fluorescence. To measure calcium in astrocytes transfected with pEGFP-β_3_ Integrin, cells were loaded with the red probe Cal-590 AM (AAT Bioquest) at 4 μM following the manufacturers’ protocol.

### Statistical analyses

Results are shown as the means ± standard error of the mean (s.e.m.) for *n* = 3 or more experiments. The results were analyzed using Kruskall-Wallis test and Dunn’s post-test. Statistical significance was set at *p* < 0.05.

## Results

### A pro-inflammatory stimulus endows primary astrocytes with Thy-1 responsive properties

**Fig. 1:**
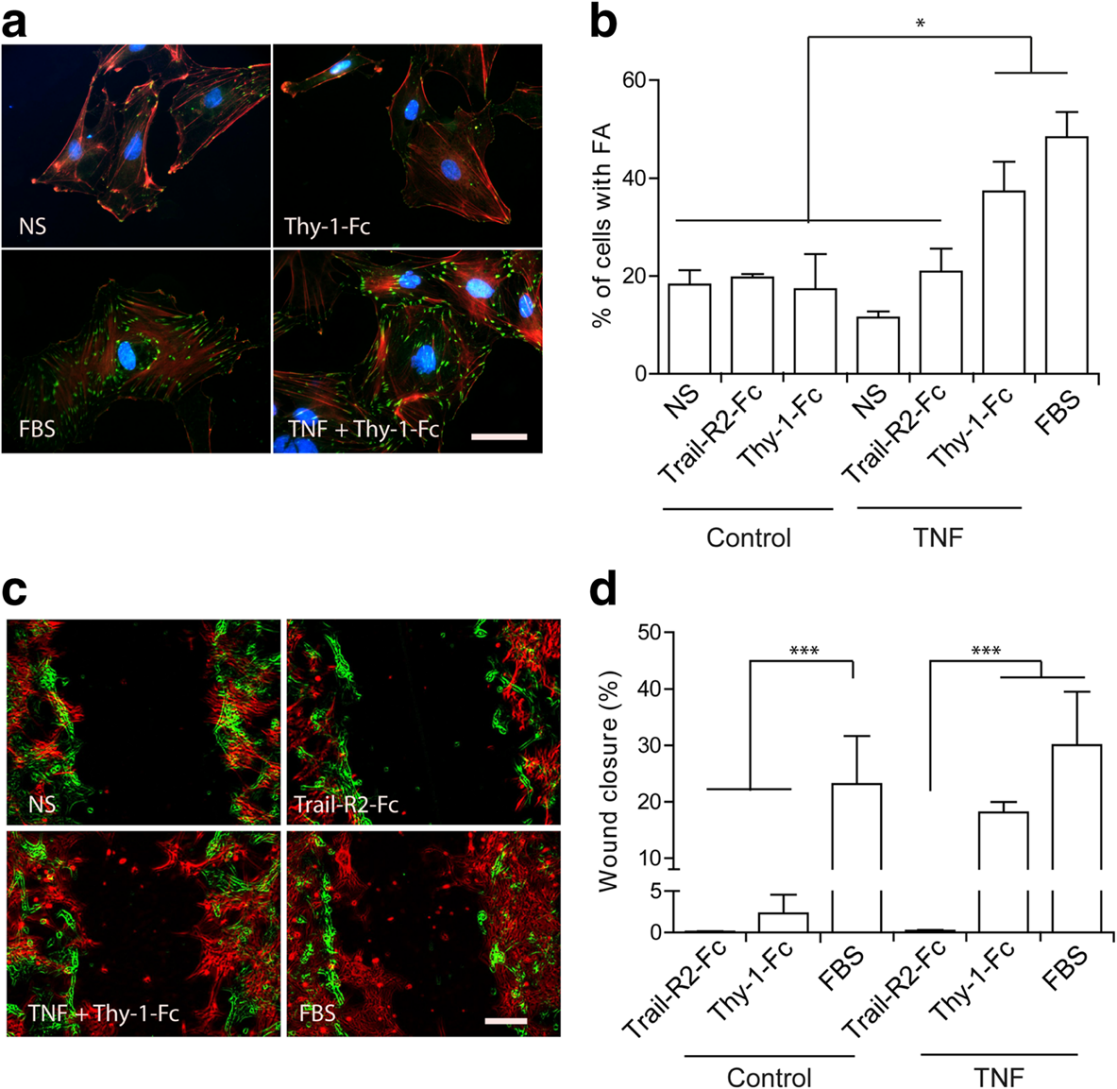
TNF treatment is required for Thy-1-induced focal adhesion formation and migration in primary astrocytes. **a** Representative images of focal adhesions (FA) in non-stimulated (NS) astrocytes or astrocytes exposed to serum (FBS) or Thy-1-Fc in the presence or absence of TNF. Trail-R2-Fc is used as a negative control. Magnification bar = 50 μm. **b** Quantification of the number of cells showing FA in primary astrocytes treated with TNF. Cells containing elongated FAs, which showed at least a 1.5-fold increase in the number of FAs compared to non-stimulated cells, were scored as cells with FAs [25]. FAs were observed in primary astrocytes treated with Thy-1 after TNF treatment, or with FBS. **c** Representative images of wound-healing assays in pseudocolor. *Green cells*: primary astrocyte cultures at 0 h. Red cells: primary astrocytes at 24 h after treatment. Magnification bar = 100 μm. **d** Wound-healing assay quantification in astrocytes treated with Trail-R2-Fc, Thy-1, or FBS for 24 h in the presence or absence of TNF. Thy-1 induces migration only in cells previously treated with TNF, whereas serum closes the wounded area in both conditions. Values shown in all graphs are the means ± s.e.m. from three independent experiments. *p < 0.05; ***p < 0.001

Thy-1 has been shown to induce of DITNC1 astrocyte migration by engaging α_V_β_3_ Integrin and Syndecan-4 [26] and triggering signaling pathways downstream of these receptors [21]. Here, the effect of Thy-1 on wild-type primary astrocytes was tested. To this end, rat astrocytes were isolated from neonatal brains. Primary cultures were prepared for FA and migration assays and starved for 30 min prior to the treatment with Thy-1-Fc, serum (FBS, positive control), or Trail-R2-Fc (negative control) [21, 26]. Cells forming FAs were monitored by vinculin staining, whereas cell migration was evaluated in a wound-healing assay. We found that, unlike FBS-treated astrocytes, the stimulation with Thy-1-Fc did not lead to FA formation and the cells were morphologically similar to non-stimulated control cells (Fig. 1a).

Because only reactive astrocytes migrate after damage to the CNS [2], primary astrocytes were thought to require activation to respond to Thy-1 stimulation. Thus, astrocytes were treated with TNF to emulate a pro-inflammatory environment and tested for the astrocyte reactivity markers GFAP and iNOS. Levels of these proteins were increased by TNF treatment (Additional file 1: Figure S1A). Astrocyte morphology also changed (Additional file 1: Figure S1B), indicating the presence of reactive astrocytes in these TNF-treated cultures.

Importantly, TNF-treated astrocytes became responsive to the addition of Thy-1-Fc, which induced formation of FAs and stress fibers (Fig. 1a). Our results show that Thy-1 stimulated only those cells previously treated with TNF (Fig. 1b) or other pro-inflammatory cytokines (Additional file 1: Figure S1C), while stimulation with serum induced FA formation in cells with or without TNF pre-treatment (Fig. 1a, b). The effect of Thy-1-Fc on astrocyte migration was also tested for these astrocytes. As expected, primary astrocytes migrated upon Thy-1 stimulation only when cells were previously treated with TNF (Fig. 1c, d). As shown for FA, serum-treated cells responded independently of TNF treatment (Fig. 1c, d). These results show that primary astrocytes assembled FA and migrated in response to FBS, but that they were insensitive to Thy-1 unless previously activated with TNF or other pro-inflammatory cytokines.

### TNF pre-treatment enables Thy-1 signaling in astrocytes

Thy-1 binding to α_V_β_3_ Integrin in DITNC1 astrocytes has been recently demonstrated to be followed by intracellular signaling events that include opening of the hemichannels Pannexin-1 and Connexin-43 and release of ATP to the extracellular milieu [21]. The ATP activates P2X7R, allowing Ca^2+^ entry and PKCα activation, followed by Syndecan-4-triggered signaling pathways [22]. The presence of these receptors as well as the activation of similar signaling pathways in wild-type rat primary astrocytes treated with TNF and stimulated by Thy-1 was therefore explored.

**Fig. 2:**
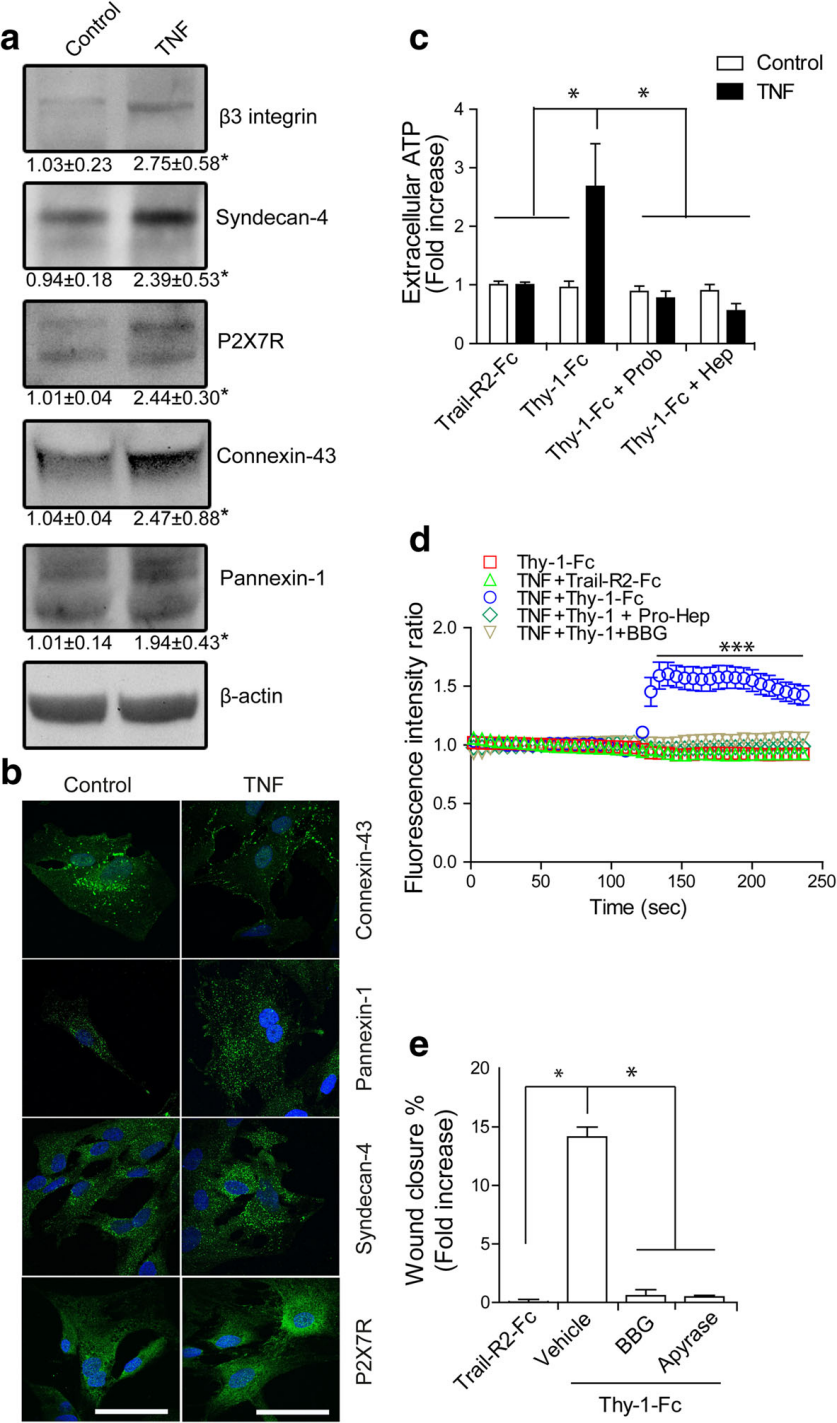
TNF pre-treatment enables Thy-1 signaling in primary astrocytes. **a** Representative western blot of β_3_ Integrin, Syndecan-4, P2X7R, Connexin-43, and Pannexin-1 after 48 h of treatment with TNF. Values indicate fold increase. **b** Localization of Connexin-43, Pannexin-1, Syndecan-4, and P2X7R in TNF-treated astrocytes and control cells (in *green*). Nuclei appear in blue. Magnification bar = 50 μm. **c** ATP measurements in the extracellular medium of primary astrocytes treated with Trail-R2-Fc or Thy-1-Fc for 10 min. Cells were previously treated, or not, with TNF for 48 h and incubated with the Connexin-blocking drug Heptanol (500 μM) and the Pannexin-blocking drug, Probenecid (1 mM). **d** Quantification of intracellular calcium levels in TNF-treated astrocytes stimulated with Thy-1-Fc and pre-treated with Heptanol and Probenecid or the P2X7R inhibitor BBG (5 μM). Trail-R2-Fc was used as a negative control. Values are 1.61 ± 0.27 for Thy-1+TNF, 1.08 ± 0.09 for Thy-1+TNF+BBG, 1.01 ± 0.02 for Thy-1+TNF+Pro-Hep, 1.06 ± 0.04 Trail-R2-Fc+TNF and 1.04 ± 0.04 for Thy-1 without TNF. **e** Wound-healing assay in rat primary astrocytes. After treatment with BBG (5 μM), Apyrase (3 UI/mL), or vehicle (PBS), cells were stimulated with Trail-R2-Fc, Thy-1-Fc for 24 h. Values shown are the means ± s.e.m. from three independent experiments. *p < 0.05

We found that pretreatment with TNF increased the levels of β_3_ Integrin, Syndecan-4, P2X7R, Connexin-43, and Pannexin-1 (Fig. 2a). β_3_ Integrin levels were also enhanced by other pro-inflammatory cytokines (Additional file 2: Figure S2). Next, the subcellular localization of these proteins was evaluated. Connexin-43 was located mainly in vesicles near the nucleus in untreated primary astrocytes but was redistributed to the cell periphery upon TNF treatment (Fig. 2b). Notably, this staining pattern was also observed in isolated cells, indicative of hemichannel rather than gap junction configuration for Connexin-43. Pannexin-1 was also redistributed; after TNF treatment, it appeared at the tip of cellular protrusions (Fig. 2b). The intensity of Syndecan-4 and P2X7R fluorescent signals increased, but neither receptor showed changes in localization (Fig. 2b).

Since hemichannel protein levels increased following TNF treatment, and because ATP is released by hemichannel opening upon Thy-1 stimulation [21, 22], ATP release to the extracellular medium in cells treated with TNF followed by Thy-1 stimulation was measured. ATP was released only when cells were pre-treated with TNF. The hemichannel blockers Probenecid and Heptanol reduced ATP release to control levels (Fig. 2c). Because extracellular ATP activates the purinergic receptor P2X7, intracellular calcium levels ([Ca^2+^]_i_) were measured following Thy-1 stimulation with and without TNF treatment. Intracellular calcium concentrations were increased by Thy-1 addition (to a maximum value of 1.61 ± 0.27) only in astrocytes pre-treated with TNF (Fig. 2d). In addition, blocking the hemichannels with a mixture of Probenecid and Heptanol or inhibiting P2X7R with Brilliant Blue G (BBG) abolished most of the [Ca^2+^]_i_ increase induced by Thy-1 stimulation, with maximum values reaching 1.01 ± 0.02 and 1.08 ± 0.09, respectively (Fig. 2d). Of note, TNF treatment per se showed no effect on ATP release or [Ca^2+^]_i_ as observed in cells treated with TNF and Trail-R2-Fc compared to those treated only with Trail-R2-Fc (Fig. 2c, d).

These results indicate that hemichannel opening, ATP release, P2X7R activation, and Ca^2+^ uptake are important steps in the signaling pathway triggered by Thy-1 in reactive primary astrocytes. In line with this, the ATP hydrolyzing enzyme Apyrase and the P2X7R inhibitor BBG both precluded Thy-1-induced migration of activated astrocytes (Fig. 2e). Therefore, and as previously reported for DITNC1 astrocytes [21, 22], these results demonstrate that ATP release and Ca^2+^ uptake via P2X7R are fundamental to the Thy-1-induced migration of astrocytes.

### Over-expression of β_3_ Integrin is sufficient to allow Thy-1-induced migration in non-activated astrocytes

**Fig. 3:**
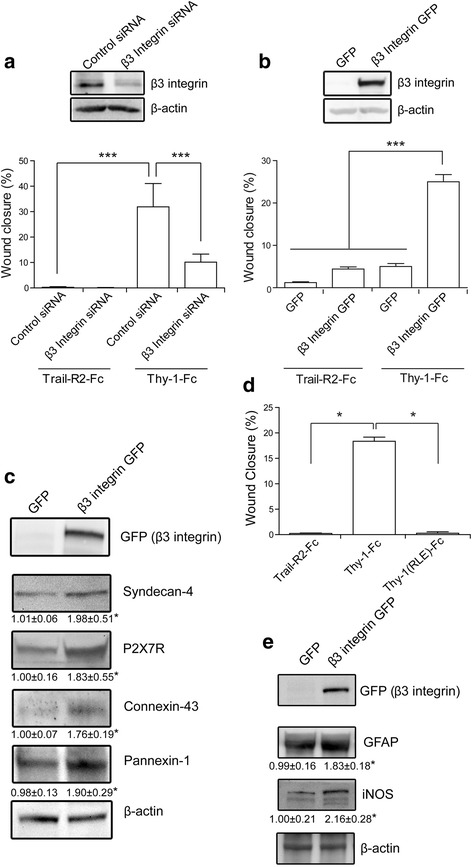
β3 Integrin expression levels affect Thy-1-induced astrocyte migration. **a** β3 Integrin knock-down reduces Thy-1-induced astrocyte migration. β3 Integrin levels and wound-healing assay quantification in β3 Integrin knock-down, TNF-treated astrocytes stimulated with Trail-R2-Fc or Thy-1-Fc. **b** Over-expression of β3 Integrin is sufficient to allow Thy-1-induced astrocyte migration. β3 Integrin levels and wound-healing assay quantification in β3 Integrin over-expressing astrocytes stimulated with Trail-R2-Fc or Thy-1-Fc. **c** Western blot of proteins of the Thy-1-induced signaling pathway in astrocytes over-expressing GFP-tagged β_3_ Integrin. **d** Wound-healing assay in TNF-treated astrocytes stimulated with Thy-1(RLE)-Fc, a Thy-1-Fc mutant that lacks the β_3_ Integrin-binding domain. **e** Western blot of markers of reactivity in astrocytes over-expressing GFP-tagged β_3_ Integrin. β actin is used as a loading control. Values shown in graphs are the means ± s.e.m. from three independent experiments. *p < 0.05; ***p < 0.001

Since β_3_ Integrin expression was enhanced after TNF treatment in primary astrocytes (Fig. 2a), and because α_V_β_3_ Integrin is a receptor for Thy-1 [21–26, 28, 47–49], the role of this Integrin in Thy-1-induced astrocyte migration was further explored. To this end, astrocytes transfected with siRNA targeting β_3_ Integrin were treated with TNF. β_3_ Integrin-silenced cells were found to contain low levels of β_3_ Integrin (not shown). These levels were kept low even after TNF treatment, in comparison to TNF-treated cells transfected with the control siRNA (Fig. 3a). The β_3_ Integrin role in Thy-1-induced migration was then evaluated in a wound-healing assay. Thy-1-induced migration was diminished in β_3_ Integrin-silenced cells compared with TNF-treated cells transfected with the control siRNA (Fig. 3a). The negative control Trail-R2-Fc had no effect on astrocyte migration (Fig. 3a).

Considering that TNF treatment induced the upregulation of many surface proteins, and that β_3_ Integrin silencing was enough to prevent Thy-1-induced migration, the effect of the β_3_ Integrin over-expression in the absence of TNF treatment was evaluated. Successful transfection of astrocytes with a vector containing pEGFP-β_3_ Integrin was corroborated by evaluating β_3_ Integrin levels with immunoblotting (Fig. 3b). Using a wound-healing assay, the β_3_ Integrin over-expression was observed to render astrocytes responsive to Thy-1, which promoted wound closure even in the absence of pro-inflammatory stimuli (Fig. 3b). Notably, β_3_ Integrin over-expression led to increased levels for many other key components of the Thy-1 response, including Syndecan-4, P2X7R, Connexin-43, and Pannexin-1 (Fig. 3c). Together, these results indicate that β_3_ Integrin over-expression is sufficient to permit Thy-1-induced migration of primary astrocytes, without the requirement for TNF treatment, and that this effect might be due to a role for β_3_ Integrin in controlling the expression of other key components of the Thy-1 signaling pathway.

To confirm that the effect of Thy-1 on wild-type primary astrocytes is mediated through the interaction of Thy-1 and α_V_β_3_ Integrin, a mutant Thy-1 carrying an amino acid substitution in the α_V_β_3_ Integrin-binding domain was used to stimulate TNF-treated astrocytes. Impaired functionality of this mutated protein has been previously reported [21, 26]. Our results show that, in contrast to wild-type Thy-1, mutant Thy-1 did not induce astrocyte migration (Fig. 3d) confirming the importance of Thy-1/α_V_β_3_ Integrin interaction in the reported effects.

Having confirmed β_3_ Integrin as a crucial mediator of Thy-1-triggered adhesion and migration, processes shown here to be limited to reactive astrocytes, and considering its upregulation in the presence of TNF, we were curious to explore whether the over-expression of this integrin had any general effects on astrocyte reactivity. The levels of the known markers of astrocyte reactivity iNOS and GFAP in pEGFP-β_3_ Integrin transfected astrocytes by western blotting were therefore evaluated. Importantly, β_3_ Integrin over-expression, but not EGFP used as a control, was found to increase the expression of these molecules (Fig. 3e), suggesting that the sole enhanced expression of this integrin was sufficient for these astrocytes to show properties of reactivity. Moreover, Thy-1 engagement of overexpressed β_3_ Integrin in astrocytes increased intracellular [Ca^2+^] and ATP levels (Additional file 3: Figures S3A and S3B). Taken together, these observations indicate that β_3_ integrin represents a key molecule in the Thy-1-induced signaling.

Importantly, these results uncover a critical role for β_3_ Integrin in primary astrocyte activation in a pro-inflammatory environment, a striking and unexpected function for this cell adhesion molecule in astrocytes.

### Thy-1 responsiveness increases in astrocytes derived from transgenic hSOD1^G93A^ mice

Neuroinflammatory diseases, such as ALS, involve cytokine release from various glial cells in the CNS [11]. Both in ALS patients and murine models of the disease, astrocytes become reactive in the spinal cord, lateral corticospinal tracts and in different brain regions before the appearance of motor symptoms [31, 50, 51]. It is therefore of interest to study the Thy-1 responsiveness of astrocytes derived from a murine model of ALS. Here, astrocytes from hSOD1^G93A^ mice and from their non-transgenic littermates were used [1, 3, 36, 41–43].

**Fig. 4:**
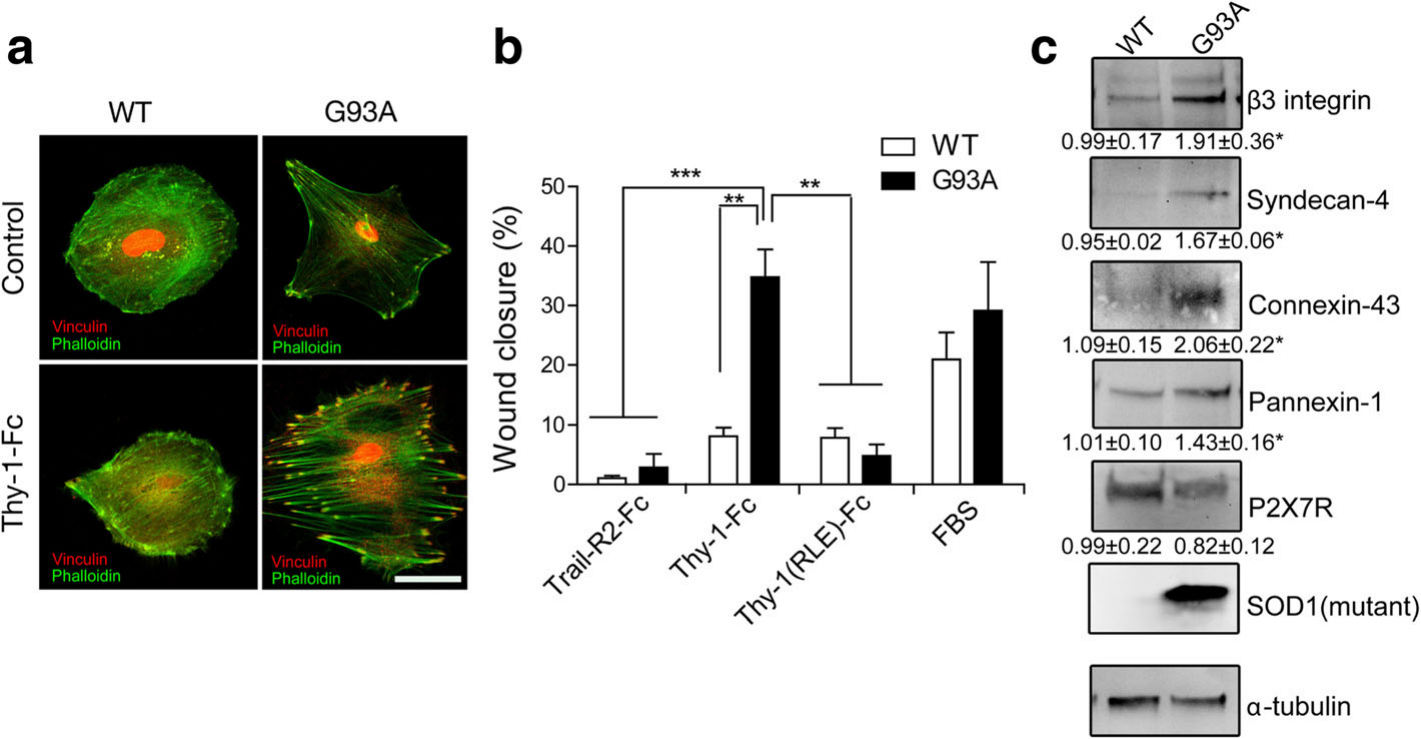
Characterization of astrocytes derived from hSOD1^G93A^ mice and from their non-transgenic littermates. **a** Representative immunofluorescence images showing FA (*red*) and stress fibers (*green*) in non-transgenic and hSOD1^G93A^-derived astrocytes in the presence or absence of Thy-1-Fc stimulation. Nuclei appear in *red*. Magnification bar = 50 μm. **b** Quantification of wound-healing assays on non-transgenic and hSOD1^G93A^-derived astrocytes treated with Trail-R2-Fc, Thy-1-Fc, Thy-1(RLE)-Fc, or serum (FBS). **c** Western blot of β_3_ Integrin, Syndecan-4, Connexin-43, Pannexin-1, P2X7R, and mutant hSOD1 protein levels in hSOD^G93A^-derived astrocytes. Values shown are the means ± s.e.m. from three independent experiments. **p < 0.01; ***p < 0.001

The activation state of these ALS astrocytes was first assessed by measuring the levels of the reactivity markers GFAP and iNOS by western blotting. The levels of both proteins were found increased when compared to astrocytes from non-transgenic animals (Additional file 4: Figure S4). In addition, vinculin and F-actin staining of non-transgenic and hSOD1^G93A^-derived astrocytes revealed differences in their FAs and actin cytoskeleton. Astrocytes from non-transgenic mice were round in shape with low levels of FAs, while hSOD1^G93A^-derived astrocytes were polygonal with better-defined actin stress fibers and fewer focal contacts (Fig. 4a, upper panels). Following Thy-1 addition, astrocytes from non-transgenic mice did not change, while hSOD1^G93A^-derived astrocytes showed an increased number of FAs and robust stress fibers (Fig. 4a, bottom panels). Next, migration was evaluated in a wound-healing assay. Only hSOD1^G93A^-derived astrocytes were found to have migrated after Thy-1-Fc stimulation (Fig. 4b). As expected, the addition of Thy-1(RLE)-Fc, which lacks the β3 Integrin-binding domain, had no effect on the migration of hSOD1^G93A^-derived astrocytes (Fig. 4b). These results confirm that hSOD1^G93A^-derived astrocytes, like TNF-activated wild-type primary astrocytes (see Fig. 1), respond to Thy-1 through α_V_β_3_ Integrin interaction, thereby increasing cell adhesion and migration.

Because increased levels of α_V_β_3_ Integrin, Syndecan-4, Connexin-43, Pannexin-1, and P2X7R had been detected in TNF-treated rat wild-type primary astrocytes, hSOD1^G93A^-derived astrocytes were tested for similar increases in the levels of these proteins. Except for P2X7R, all of these proteins were found to be upregulated in these cells (Fig. 4c).

Together, these results confirm that hSOD1^G93A^-derived astrocytes are reactive, show increased expression of surface proteins involved in Thy-1-induced signaling, and enhanced adhesion and migration after Thy-1-Fc stimulation. It is intriguing that, despite their early postnatal origin (P1-P2 stages), hSOD1^G93A^-derived astrocytes showed properties of reactive astrocytes after only 4 weeks in culture, suggesting that despite their early origin, these astrocytes were already primed to become reactive.

## Discussion

Inflammatory cytokines are widely considered causative in triggering astrocyte reactivity [52]. Here, we show that wild-type primary astrocytes respond to Thy-1 by adhering and migrating only under inflammatory conditions (+TNF). We also show that in primary astrocytes, Thy-1 induces similar signaling pathways to those reported for DITNC1 astrocytes, namely, hemichannel opening, ATP release, activation of P2X7R, Ca^2+^ entry, early cell adhesion, and finally, cell polarization and migration [21, 26, 53, 54]. In addition, hSOD1^G93A^-derived astrocytes respond to Thy-1 without any requirement for additional cytokines. Of note, ectopically expressed β_3_ Integrin increases the levels of other important players of the Thy-1-induced signaling pathways, including Syndecan-4 and the Connexin-43/Pannexin-1 hemichannels, as well as markers of reactive astrocytes, such as iNOS and GFAP. Furthermore, over-expression of β_3_ Integrin is sufficient to render astrocytes responsive to Thy-1. Therefore, we propose that β_3_ Integrin plays a preponderant role in determining Thy-1-induced effects in astrocytes and possibly also in controlling astrocyte reactivity.

After injury to the CNS, microglia generate a pro-inflammatory environment by liberating TNF [11]. This has been shown to enhance the expression of integrins [55] and cell adhesion molecules, such as ICAM-1, VCAM-1, and E-selectin [52, 56, 57]. In our studies, TNF induced astrocyte reactivity, increasing the expression levels of reported markers of astrocytosis as well as the Thy-1 receptors α_V_β_3_ Integrin and Syndecan-4 [25, 28, 58]. Thy-1 receptor upregulation enables cells to respond to the ligand and thereby initiate astrocyte adhesion and migration. These findings suggest that a pro-inflammatory environment is required for astrocytes to become responsive to neuronal signals such as Thy-1. The low levels of β_3_ Integrin observed in primary astrocytes may explain the unresponsiveness of these cells to Thy-1. In this context, it is of interest that TNF also induces α_V_β_3_ Integrin expression, along with a α_V_β_3_ Integrin-dependent cell migration, in endothelial cells [44]; this indicates that increased α_V_β_3_ Integrin expression is relevant to migration under pro-inflammatory conditions in other cellular models.

In the present study, the application of TNF to primary astrocytes leads to an increased expression of α_V_β_3_ Integrin, Syndecan-4, P2X7R, Connexin-43, and Pannexin-1. All of these molecules have previously been shown to be required for Thy-1-induced cell migration [21]. Others have reported that Connexin-43 hemichannnels are activated by treating astrocytes with a mixture of TNF and IL-1β. This leads to neuronal death, an effect not observed in astrocytes lacking Connexin-43 [59]. Moreover, prenatal exposure to LPS, a well-known pro-inflammatory agent, induces activation of astrocytes with increased levels of Connexin-43 and Pannexin-1 [4]. Additionally, treating astrocytes with TNF for only 3 h induces expression of Connexin-43 hemichannels [60]. These results suggest that primary astrocytes exposed to a pro-inflammatory environment upregulate surface receptors involved in the Thy-1-Integrin-induced signaling pathways.

ATP release from hemichannels activated P2X7R and thus increased intracellular calcium, only after TNF treatment. ATP release and P2X7R activation has been previously shown to be involved in Thy-1-induced astrocyte responses, first inducing cell adhesion [22] and subsequently migration [21]. Of note, increased levels of ATP have been associated with brain damage in humans. Patients with radiation-induced brain injury show high levels of extracellular ATP, IL-6, and TNF in cephalorachidian fluid [61]. In addition, exposure of primary astroglial cultures to radiation increases extracellular levels of ATP, IL-6, and TNF, unless P2X7R is inhibited or silenced [61]. This suggests an important role for ATP and P2X7R in the activation of glial cultures and progression of brain injuries. Recently, increased expression of P2X7R and Connexin-43 has been linked to astrogliosis in autoimmune encephalomyelitis, where the increase of P2X7R coincided with the appearance of astrocyte reactivity markers, such as GFAP and S100β. The addition of P2X7R blockers led to a reduction in astrogliosis markers [20], suggesting that the increase in P2X7R is involved in astrocyte reactivity. In line with this, published evidence indicates that P2X7R over-expression leads to microglia activation [62, 63]. In the present study, P2X7R protein levels increased after TNF treatment, while the inhibition of P2X7R and the apyrase-catalyzed reduction of extracellular ATP both decreased Thy-1-induced astrocyte migration. However, it remains to be determined whether *P2X7R* is a direct or an indirect target of TNF.

The increase in [Ca^2+^]_i_ induced by Thy-1 stimulus is necessary for astrocyte migration. Ca^2+^ influx and Ca^2+^ release from intracellular stores via stimulation of IP3R participate in Thy-1-induced astrocyte migration, since P2X7R silencing as well as the addition of IP3R inhibitors prevent the migration of astrocytes with a reactive phenotype [21]. In addition, the calcium-chelator BAPTA-AM blocks Thy-1-induced hemichannel opening and ATP release, two key steps in the process of Thy-1-induced DITNC1 astrocyte migration [21]. In agreement with our observations, Fang and coworkers published that Orexin, a neuropeptide that evokes responses similar to those triggered by Thy-1 in astrocytes, induces migration in rat astrocytes in a Ca^2+^-dependent manner, which can be blocked by either the addition of BAPTA-AM or an IP3R blocker, such as 2-APB [64], supporting the notion that increased [Ca^2+^]_i_ is necessary for astrocyte migration.

In primary astrocytes, TNF treatment is required for the Thy-1 response; however, TNF by itself neither induces migration nor increases Ca^2+^ levels (Figs. 1c, d and 2d), but rather promotes changes in protein expression to generate a migratory phenotype responsive to Thy-1. Of note, α_V_β_3_ Integrin, Syndecan-4, and P2X7 receptors trigger parallel and different signaling pathways [21]; thus, although elevated [Ca^2+^]_i_ is required for Thy-1-stimulated astrocyte migration, it is not expected to be sufficient alone to induce the migratory phenotype, which results from more complex and interconnected signaling cascades.

Thy-1 elicits responses by engaging its receptors α_V_β_3_ Integrin and Syndecan-4 [21, 22, 26]. Interestingly, β_3_ Integrin over-expression was sufficient to enable primary astrocytes to respond to Thy-1, thereby showing an effect similar to that of TNF treatment. Others have reported an increased cell migration, invasion and tumorigenesis of cancer cells over-expressing β_3_ Integrin following stimulation with osteopontin [65], an extracellular matrix protein, which is upregulated and secreted after TNF treatment, and which induces directional astrocyte migration in an α_V_β_3_ Integrin-dependent manner [18, 19]. This is in line with our findings, since it indicates that increased levels of β_3_ Integrin protein make cells more responsive to external cues. However, over-expression of β_3_ Integrin alone does not induce tumorigenesis in the absence of osteopontin [65]. This is paralleled by our own observation that in the absence of Thy-1, migration was not increased in β_3_ Integrin over-expressing astrocytes, confirming that elevated levels of β_3_ Integrin render astrocytes sensitive to the neuronal Thy-1, rather than increasing cell autonomous migration. The intriguing possibility that increased α_V_β_3_ Integrin expression might guide reactive astrocytes to regions rich in “damage” signals, allowing them to interact with neuronal Thy-1 in the damaged zone, awaits further studies.

Furthermore, β_3_ Integrin appears to control the expression of other proteins involved in the Thy-1 response. It has been reported that α_V_β_3_ Integrin acts upstream of NF-κB in several cell types [66–68] and that tyrosine 759 of β_3_ Integrin is required for NF-κB activation [68], suggesting that this pathway, or a similar one, might be activated upon over-expression of β_3_ Integrin. Like α_V_β_3_ Integrin, the expression of the Thy-1 co-receptor, Syndecan-4, is also increased in an NF-κB-dependent manner when EAhy926 cells are treated with TNF [69]. It is possible that NF-κB activation might increase expression of the surface proteins reported in the present study in response to TNF. In hepatocytes from acute-on-chronic liver failure, increased levels of NF-κB are associated with increased expression of Connexin-43, but not Connexin-26 or Connexin-32 [70]. In isolated aorta sections, Connexin-43 levels are increased downstream of NF-κB and ERK1/2 activation [71]. Additionally, IL-8 regulates α_V_β_3_ Integrin expression in a PI3K/Akt/NF-κB-dependent manner in MDA-MB-231 cancer cells [45]. Similarly, when glioblastoma multiforme cells are treated with the phorbol ester PMA, NF-κB induces the expression of α_V_β_3_ Integrin along with fibronectin and vitronectin [72]. This suggests a possible role for NF-κB in upregulating the expression of components in the Thy-1 activated pathway. Consequently, β_3_ Integrin could be placed upstream or downstream of NF-κB. Considering that β_3_ Integrin over-expression is sufficient to increase the expression of reactive astrocyte markers and to render astrocytes sensitive to Thy-1 stimulation in the absence of TNF, and since β_3_ Integrin silencing precludes the effect of Thy-1 in TNF-treated cells, we propose that β_3_ Integrin expression can control astrocyte reactivity. Whether it does so by activating NF-κB remains to be assessed.

To place our findings into a patho-physiological context, astrocytes were analyzed from the hSOD1^G93A^ ALS mouse model. In this model, astrocytes are reported to be reactive before the onset motor symptoms, and the degree of reactivity correlates with the neurodegenerative process [50]. Elevated expression levels of GFAP, Vimentin, and CD44 in tissue sections from post-mortem spinal cord in ALS patients also indicate astrocyte reactivity [36]. Here, Thy-1 is shown to induce adhesion and migration of hSOD1^G93A^-derived astrocytes without the need of prior TNF treatment. As might be expected, Connexin-43 levels substantially increased in these cells (Fig. 4c; see also [3]). For P2X7R, modest levels of expression are associated with increased ALS severity, as well as increased astrocyte and microglia reactivity [1]. Consistent with this, we observed low levels of P2X7R in hSOD1^G93A^ astrocytes, suggesting that these levels are sufficient for Thy-1-induced migration. Therefore, astrocytes derived from hSOD1^G93A^ mice are reactive and responsive to Thy-1. Notably, unlike P2X7R, Pannexin-1, Syndecan-4, and α_V_β_3_ Integrin expression levels were increased. Such alterations have not been associated with ALS to date, and further studies are required to clarify the specific contribution of each of these proteins.

## Conclusions

We show for the first time that primary astrocytes must be primed by exposure to a pro-inflammatory environment before they can change their shape, adhesion, and migratory properties in response to neuronal Thy-1. β_3_ Integrin was identified as a fundamental mediator of Thy-1-induced migration, not only as a receptor, but also as a regulator of the signaling pathway downstream of the Thy-1 receptors. In addition, we propose β_3_ Integrin as a key target to control astrocyte reactivity. Finally, a novel association of Pannexin-1, Syndecan-4, and β_3_ Integrin with ALS is here uncovered. Therefore, our results support Thy-1 and β_3_ Integrin/Syndecan-4 as potential targets for the clinical management of neurodegenerative diseases involving inflammatory processes.

## Additional files

Additional file 1: Figure S1. A) Representative western blot of astrocyte reactivity markers GFAP and iNOS from wild-type rat astrocytes treated or not with TNF. B) Representative images of GFAP immunofluorescence from rat primary astrocytes treated or not with TNF. Magnification bar = 50 μm. C) Quantification of rat primary astrocytes harboring FAs in control cells or cells treated with IL-1β, IL-6, or TNF, and stimulated or not with Thy-1-Fc or FBS. Values shown are the means ± s.e.m. from three independent experiments. ***p < 0.001. (PDF 2294 kb)

Additional file 2: Figure S2. Representative western blot of β3 Integrin from rat astrocytes treated with Il-6, IL+db cAMP, IL-1β, IL-1β+db cAMP, or IFN-γ. (PDF 77 kb)

Additional file 3: Figure S3. β3 Integrin over expression permits Thy-1-induced signaling. A) Quantification of intracellular calcium levels in rat primary astrocytes transfected with pEGFP-β3 Integrin or pEGFP and stimulated with Thy-1-Fc. Trail-R2-Fc was used as a negative control. Cells were starved for 30 min. The arrow indicates the addition of Thy-1. B) Extracellular ATP measurement of primary astrocytes transfected with pEGFP-β3 Integrin or pEGFP, and treated with Trail-R2-Fc or Thy-1-Fc for 10 min. Cells were previously starved for 30 min. Values shown in the graph are the mean ± s.e.m. from three independent experiments. *p < 0.05. (PDF 174 kb)

Additional file 4: Figure S4. Representative western blot of the markers of astrocyte reactivity GFAP and iNOS from non-transgenic and hSOD1^G93A^-derived astrocytes. β-actin is a loading control. (PDF 166 kb)

## Abbreviations

[Ca^2+^]i: Intracellular calcium concentrations
ALS: Amyotrophic lateral sclerosis
ATP: Adenosine triphosphate
BBG: Brilliant Blue G
CNS: Central nervous system
FA: Focal adhesion
GFAP: Glial fibrillar acidic protein
P2X7R: P2X7 receptor
SOD1: Super oxide dismutase 1
TNF: Tumor necrosis factor
