# Supplementary figures and images for: αVβ3 Integrin regulates astrocyte reactivity
Source: J Neuroinflammation. 2017 Sep 29;14:194. doi: 10.1186/s12974-017-0968-5 (PMC5622429; doi:10.1186/s12974-017-0968-5)

# Additional File: Figure S1

A Rat primary astrocytes

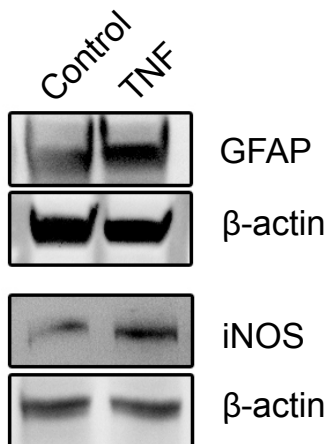

B

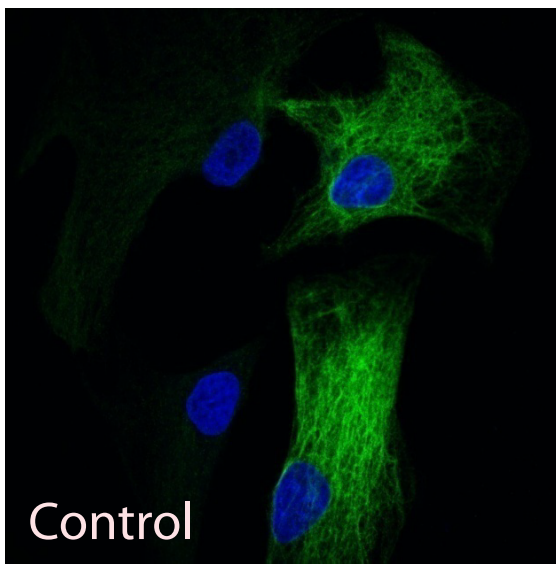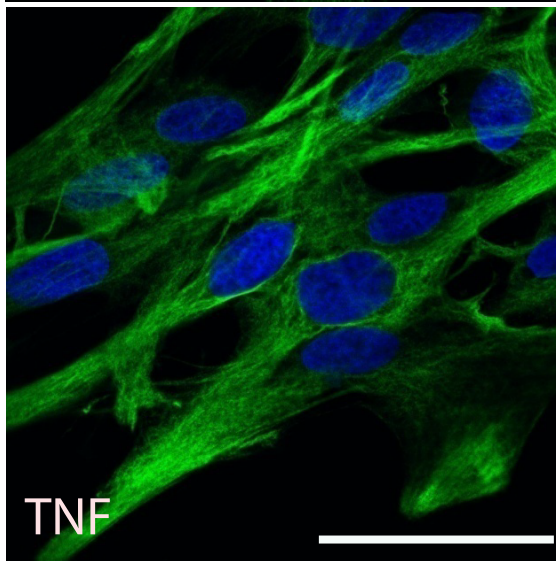

C

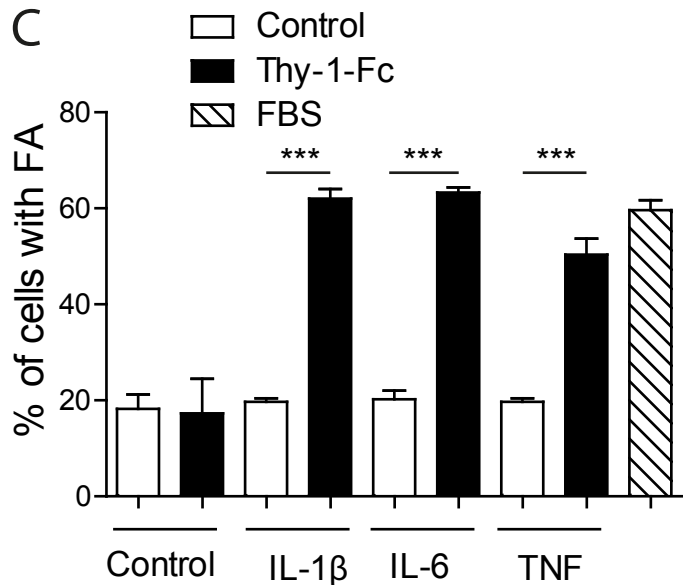

Supplement: Supplementary file 1 — A) Representative western blot of astrocyte reactivity markers GFAP and iNOS from wild-type rat astrocytes treated or not with TNF. B) Representative images of GFAP immunofluorescence from rat primary astrocytes treated or not with TNF. Magnification bar = 50 μm. C) Quantification of rat primary astrocytes harboring FAs in control cells or cells treated with IL-1β, IL-6, or TNF, and stimulated or not with Thy-1-Fc or FBS. Values shown are the means ± s.e.m. from three independent experiments. ***p < 0.001. (PDF 2294 kb) [file 12974_2017_968_MOESM1_ESM.pdf]

## Additional File: Figure S2

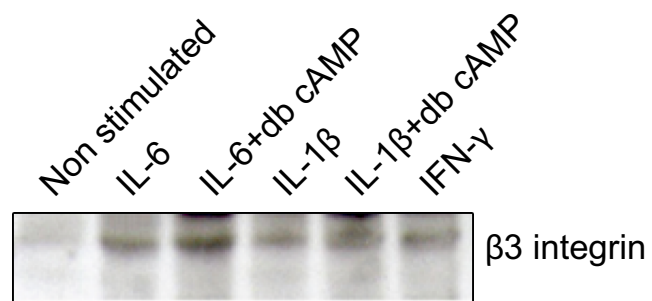

Supplement: Supplementary file 2 — Representative western blot of β3 Integrin from rat astrocytes treated with Il-6, IL+db cAMP, IL-1β, IL-1β+db cAMP, or IFN-γ. (PDF 77 kb) [file 12974_2017_968_MOESM2_ESM.pdf]

# Additional File: Figure S3

A

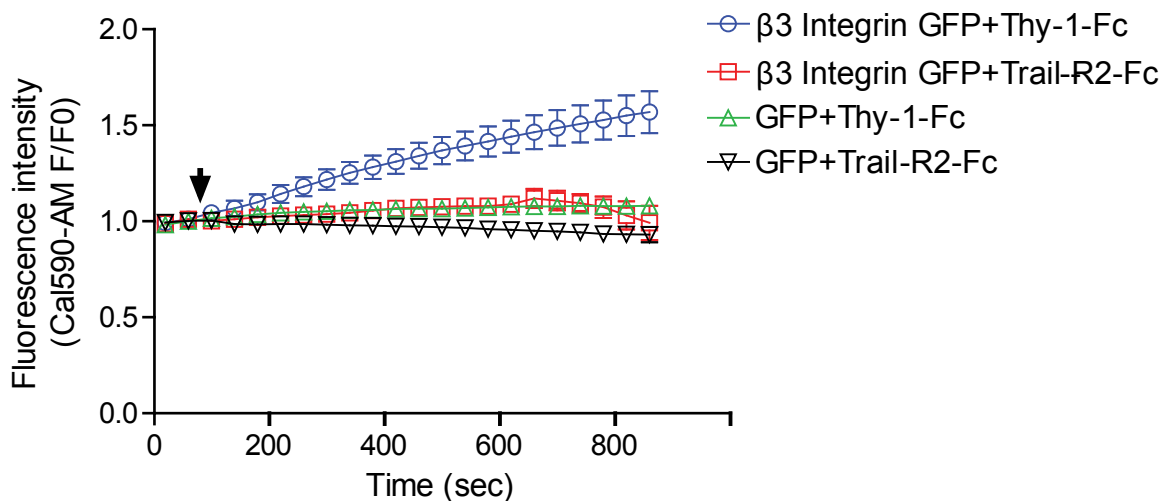

B

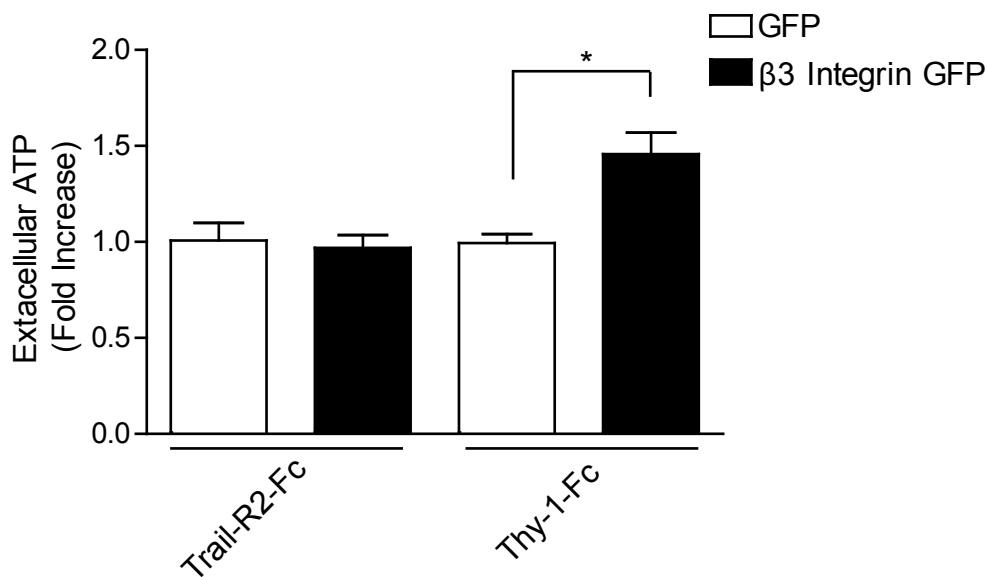

Supplement: Supplementary file 3 — β3 Integrin over expression permits Thy-1-induced signaling. A) Quantification of intracellular calcium levels in rat primary astrocytes transfected with pEGFP-β3 Integrin or pEGFP and stimulated with Thy-1-Fc. Trail-R2-Fc was used as a negative control. Cells were starved for 30 min. The arrow indicates the addition of Thy-1. B) Extracellular ATP measurement of primary astrocytes transfected with pEGFP-β3 Integrin or pEGFP, and treated with Trail-R2-Fc or Thy-1-Fc for 10 min. Cells were previously starved for 30 min. Values shown in the graph are the mean ± s.e.m. from three independent experiments. *p < 0.05. (PDF 174 kb) [file 12974_2017_968_MOESM3_ESM.pdf]

# Additional File: Figure S4

Mouse primary  
astrocytes

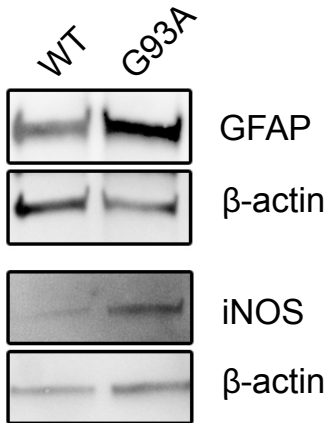

Supplement: Supplementary file 4 — Representative western blot of the markers of astrocyte reactivity GFAP and iNOS from non-transgenic and hSOD1G93A-derived astrocytes. β-actin is a loading control. (PDF 166 kb) [file 12974_2017_968_MOESM4_ESM.pdf]
